# Psychometric properties of the Clinical Assessment Interview for Negative Symptoms (CAINS) in patients with depression and its relationship to affective symptoms

**DOI:** 10.1186/s12991-023-00474-x

**Published:** 2023-10-26

**Authors:** Johan Bengtsson, Parya Rad, Martin Cernvall, Robert Bodén

**Affiliations:** 1 Department of Medical Sciences, Psychiatry, Uppsala University, Entrance 10, Uppsala University Hospital, 751 85 Uppsala, Sweden; 2 Uppsala University Hospital, Uppsala, Sweden

**Keywords:** Validation, Motivation, Pleasure, Expression, Affective disorders, Transdiagnostic

## Abstract

**Background:**

There is a conceptual overlap between negative and depressive symptoms, requiring further exploration to advance the understanding of negative symptoms. The aim of this study was to examine psychometric properties of the Clinical Assessment Interview for Negative Symptoms (CAINS) in patients with depression, and to explore the relationship between the negative and affective symptoms domains.

**Methods:**

Fifty-one patients with a depressive episode were included and interviewed with the CAINS and the Brief Psychiatric Rating Scale—Expanded (BPRS-E). Self-reported depressive symptoms were collected with the Montgomery-Asberg Depression Rating Scale (MADRS-S). Inter-rater agreement, internal consistency and validity measures were examined, as were correlations between negative and affective symptoms.

**Results:**

The intraclass correlation for the CAINS motivation and pleasure subscale (CAINS-MAP) was 0.98 (95% CI 0.96–0.99) and that for the expressional subscale (CAINS-EXP) was 0.81 (95% CI 0.67–0.89). Cronbach’s alpha was 0.71 (95% CI 0.57–0.82) for the CAINS-MAP and 0.86 (95% CI 0.79–0.92) for the CAINS-EXP. The correlation with the negative symptoms subscale of the BPRS-E was 0.35 (*p* = 0.011, blinded/different raters) or 0.55 (*p* < 0.001, not blinded/same rater). The CAINS-MAP correlated with the affective symptoms subscale of the BPRS-E (*r *= 0.39, *p* = 0.005) and the MADRS-S total score (*r *= 0.50, *p* < 0.001), but not with anxiety symptoms.

**Conclusions:**

Negative symptoms in depression can be assessed with the CAINS with good inter-rater agreement and acceptable internal consistency and validity. There are associations between negative and depressive symptoms that call for further exploration.

**Supplementary Information:**

The online version contains supplementary material available at 10.1186/s12991-023-00474-x.

## Background

Negative symptoms, including anhedonia, avolition, asociality, alogia and blunted affect, are usually considered to be part of schizophrenia [1]. There has been growing interest in the negative symptoms domain of schizophrenia in the recent decades [2]. One issue of special interest has been the conceptual overlap between negative symptoms and depressive symptoms and consensus has been reached on the importance of distinguishing between them [3]. This is relevant partly because of the need to diagnose depression in patients with schizophrenia [4], but also because this line of research can promote better characterization of the concept of negative symptoms [2, 3, 5]. This is crucial for several fields: neurobiological research on mechanistic pathways [6, 7], studying the influence of pharmacological effects on negative symptoms [8], but also because there are findings showing that clinicians tend to assign the same information different meanings when they already know a patient’s diagnosis, thus introducing a risk of bias in defining the concept [9]. Thus, if a patient has a schizophrenia diagnosis the bias may lean towards labelling certain behaviours and symptoms as negative symptoms, whereas if the patient has a depressive disorder the similar phenomenology might be interpreted and labelled as depressive symptoms.

To address these issues, some studies have investigated negative symptoms in patients with depression, or in mixed samples of depression or schizophrenia. The emerging picture is that patients with depression often exhibit some level of negative symptoms [10, 11]. When investigating mixed samples, there is indeed an overlap, but it may be possible to distinguish negative symptoms from depressive symptoms [12–14]. In other words, the overlap is incomplete, and expressional features have been suggested as a possible marker to distinguish between the two constructs in mixed samples [14]. In studies of individuals with a primary diagnosis of schizophrenia, it has also been suggested that it is possible to distinguish between negative symptoms and depressive symptoms, albeit still acknowledging that the phenomenology is overlapping [15]. This is corroborated by findings that expressional features such as motor retardation, blunted affect and alogia have been found to have the weakest correlations with depressive symptoms in a large sample of patients with schizophrenia [5]. A challenge to this line of research is also the diversity of the depression diagnosis [16].

Several rating scales have been developed to assess negative symptoms, though mainly in patients with schizophrenia [8]. The Clinical Assessment Interview for Negative Symptoms (CAINS) was developed to address conceptual and psychometric limitations of existing instruments [17]. The CAINS is a semi-structured interview estimating the patient’s motivation for and pleasure in social interactions and engagement in social/recreational activities and working/studying. Emotional expression is assessed through observations during the interview. As the CAINS is now one of the recommended tools for assessing negative symptoms in schizophrenia [8], there is a need for studies in patients with depression, to validate if it can be used in this population as well. This would enable further exploration of the overlap between negative symptoms and depressive symptoms, both in depression only but also in transdiagnostic samples. One earlier study using the CAINS in patients with depression and schizophrenia concluded that reduced expression can be used to distinguish between the two diagnostic constructs [14]. As the overlap between negative symptoms and depressive symptoms might complicate the delineation of negative symptoms, the possibility to use the same rating scale transdiagnostically may be of help for disentangling the constructs [18].

The aim of this study was to examine inter-rater agreement, internal consistency and validity measures of the CAINS in patients with depression. A further aim was to explore the relationship between the negative symptoms domain and the depressive and anxiety symptoms domains, as well as measures of treatment resistance, global clinical impression, functional and cognitive features. Our hypothesis was that inter-rater agreement and consistency measures would resemble earlier reports from populations of patients with schizophrenia. Regarding validity, we hypothesized that established assessments of negative symptoms would correlate to the CAINS also in our population of patients with depression. We also hypothesized that there would be correlations between negative and affective symptom domains, indicating an overlap between these.

## Methods

### Participants

The sample included 51 outpatients diagnosed with major depressive disorder or bipolar disorder, with a current depressive episode. The diagnoses were confirmed using the Mini-International Neuropsychiatric Interview [19]. All participants had participated in a transdiagnostic randomized controlled trial of repetitive transcranial magnetic stimulation for treating negative symptoms in schizophrenia and depression [20] or an add-on brain imaging study [21]. The current study included subjects from these studies who had depression. Inclusion criteria were: (1) having a diagnosis of a major depressive disorder or bipolar disorder, with an ongoing depressive episode, as defined in the 10th revision of the International Statistical Classification of Diseases and Related Health Problems, (2) age 18–59 years and (3) scoring ≤ 40 on the Motivation and Pleasure Scale-Self Report (MAP-SR) [22]. The last criterion was due to the design of the aforementioned trial, where a certain level of negative symptom burden was required (see also the "Discussion" section). Exclusion criteria were (1) a severe medical condition, (2) epilepsy, (3) metal or cochlear implant in the head, (4) changes in medication during the preceding month, (5) current substance use disorder (illicit drugs or alcohol), (6) regular treatment with benzodiazepines, (7) pregnancy and (8) insufficient proficiency in Swedish. Patients were recruited from the affective disorder clinic at Uppsala University Hospital in 2016–2020.

### Assessments

The CAINS is a semi-structured interview consisting of 13 items divided into two parts: the CAINS motivation and pleasure subscale (CAINS-MAP) and the CAINS expression subscale (CAINS-EXP). The CAINS-MAP comprises nine items assessing the patient’s motivation and pleasure in social interactions and engagement in social and recreational activities/working/studying. In the CAINS-EXP, the interviewer assesses the patient’s facial and vocal expressions, expressive gestures and quantity of speech in four items. All items are scored 0–4, where 0 is described as ‘No impairment’ and 4 as ‘Severe deficit’.

The Brief Psychiatric Rating Scale—Expanded (BPRS-E) is one of several rating scales widely used for assessing negative symptoms [8]. It is a clinician-rated global rating scale where all 24 items are scored 1–7, with 1 being ‘Not present’ and 7 is ‘Extremely severe’. The items yield a relatively comprehensive description of major psychiatric symptom characteristics. In earlier studies, several subscales of the BPRS have been used [23]. In this study, we opted for the negative symptoms subscale consisting of the sum of the items *Blunted affect*, *Emotional Withdrawal* and *Motor retardation*. The positive symptoms subscale used in this study consisted of the sum of the items *Suspiciousness*, *Hallucinations* and *Unusual Thought Content*. For the affective symptoms subscale, we used the sum of the items *Depression*, *Suicidality* and *Guilt* [23]. We also analysed anxiety symptoms using the sum of the two items *Anxiety* and *Tension*.

The Clinical Global Impression (CGI) is a clinician-rated Likert-type scale 1–7, with 1 being ‘normal or not at all ill’ and 7 being ‘severe’ [24, 25].

The self-assessment instrument Montgomery-Asberg Depression Rating Scale (MADRS-S) comprises nine items that are scored from 0 (no symptoms) to 6 (very severe) [26]. The MADRS-S has been validated in Swedish patients with depression in comparison with the Beck Depression Inventory [26]. In addition to using the total sum of MADRS-S, we analysed the sum of the items *Lassitude* and *Inability to feel*, calling this entity ‘MADRS-S anhedonia’. There is no established consensus on a validated factor structure for assessing anhedonia using the MADRS-S, but there are several earlier approaches, and they all include at least one of these items [27–30]. Since we also used the total MADRS-S score, we opted for the aforementioned combination of items, in order to avoid redundancy. We also examined anxiety symptoms with the item *Inner tension*.

The Maudsley Staging Method (MSM) is a tool used to stage severity of treatment resistance in depression [31]. The MSM assesses duration (scored 1 to 3) and severity (scored 1 to 5) of the current depressive episode. The MSM also assesses number of antidepressant treatment failures (categorized and scored 0 to 5), augmentation (scored 0 or 1), and electroconvulsive therapy (scored 0 or 1) in the current episode*.* It was included because highly treatment-resistant patients might differ in regard to both motivational and expressional features. The MSM has not been explicitly validated in Swedish patients with depression, but has shown predictive value for treatment-resistant depression [31].

The EuroQol Group Visual Analogue Scale (EQ-VAS) is a self-rated instrument where the patient rates their overall health on a visual analogue scale. The endpoints of the scale are 0 ‘The worst health you can imagine’ and 100 ‘The best health you can imagine’ [32].” The EQ-VAS has been used in Swedish populations of the general population including groups with suspected depression [33–35].

The Sheehan Disability Scale (SDS) [36, 37] is a self-rated instrument measuring functional impairment through a total of five items. In the SDS, the patient assesses the degree to which symptoms have affected their work/studies, social life and family interactions. The score ranges from 0 to 10, with 10 being ‘Very much’.

The Alcohol Use Disorders Identification Test (AUDIT) assesses alcohol drinking habits and consequences and was used to gather the clinical characteristics of the sample [38]. The psychometric properties of the Swedish version of the AUDIT have been investigated [39].

Cognitive functioning was assessed with two tests. In the Animal Naming Test, which assesses semantic verbal fluency, the subject is asked to name as many animals as possible during one minute [40]. In the Digit Symbol Substitution Test (DSST) [41], the subject does coding exercises and their processing speed is measured. In both these tests, a higher score indicates higher cognitive functioning.

### Procedures

The authors assert that all procedures contributing to this work comply with the ethical standards of the relevant national and institutional committees on human experimentation and with the Helsinki Declaration of 1975, as revised in 2008. All procedures were approved by regional ethical review board at Uppsala University. All participants provided written informed consent. The raters were a psychiatrist (RB), two residents in psychiatry (JB and PR) and a clinical psychologist (MC). All were trained in administration of the CAINS under the supervision of the most senior psychiatrist (RB). For training, nine videotaped interviews of both the CAINS (some of which was provided by the original developers through https://redcap.med.upenn.edu/surveys/?s=3794388R39RYEDC7&referrer=120) and the BPRS were watched and assessed by the raters. The inter-rater agreement after training was high for both the CAINS and the BPRS, including the subscales (all intraclass correlation coefficients (ICCs) above 0.80, see Additional file 1: Table S1). In the study, the participants were interviewed with the CAINS and the BPRS, in that order, while being videotaped. These interviews were performed by either author JB or RB. A second rater watched the taped interviews in the opposite order. This rater could be any of the authors JB, PR or MC (JB only if he had not performed the live interview). The video raters were given instructions to avoid rewinding or pausing the recording, in so far as possible. In order to enable a blinded design where assessment of one scale was blinded in assessment of the other, the CAINS scores from the live interview and the BPRS scores from the video rating were used in the validity analyses. This procedure was due to the importance of not being aware of the result from the first interview (CAINS) when conducting the second (BPRS), as recommended by validation guidelines [42].

The negative subscale of the BPRS was used as the reference test to analyse construct validity. Correlations with other affective domains were computed using the BPRS affective subscale, the total score of the MADRS-S and the MADRS-S anhedonia items (*Lassitude* and *Inability to feel*). We also analysed the anxiety symptom items of the BPRS and the MADRS-S. The rater who carried out the CAINS live rating also scored the CGI. The EQ-VAS questionnaire was used to assess correlation with self-rated health. The SDS was used to calculate the correlation between the CAINS and disability. Further, the Animal Naming Test and the DSST were administered by a research nurse; these tests were used to determine cognitive function. The patients were also assessed regarding treatment resistance using the MSM.

### Statistics

All data were visually inspected for normality distribution, using histograms. For variables where the distribution was somewhat skewed, the mean and median values were compared. As these values did not differ substantially, means are reported throughout. The ICC was used to estimate inter-rater agreement (two-way random effects, consistency) [43]. Cronbach’s alpha with a 95% confidence interval (CI) was used to calculate internal consistency [44]. Scatterplots were visually inspected for linearity between correlation variables. Pearson’s correlation coefficients were used in the validity analyses. Differences in correlations between ratings of the CAINS and blinded versus unblinded ratings of the BPRS negative symptoms subscale were investigated using the r.test function in the psych package for R (Revelle, W. 2020. psych: Procedures for Personality and Psychological Research. Northwestern University, Evanston, https://CRAN.r-project.org/package=psych. R package version 2.0.8). JASP (JASP Team 2022. Version 0.11.1) was used for the remaining statistical analyses.

## Results

### Demographic and clinical characteristics

Descriptive data are presented in Table 1. About half of the participants were female and a vast majority had unipolar depression as their primary diagnosis. More than a third had a comorbid anxiety disorder and just over a fifth had been diagnosed with attention deficit hyperactivity disorder. Serotonin-norepinephrine reuptake inhibitors were the type of medication most frequently prescribed. A quarter of the participants were treated with mood stabilizers. Mean CAINS total score was 29.2 (standard deviation (SD) 7.7) and mean BPRS total score was 46.6 (SD 6.3). Mean MADRS-S total score was 29.9 (SD 7.7).

**Table 1:** Demographic and clinical characteristics of the sample (*n* = 51)

	*N*	%
Female/male	27/24	53/47
Primary diagnosis unipolar depression	46	90
Primary diagnosis bipolar depression	5	10
Comorbidity		
Anxiety disorders	21	37
Autism spectrum disorders	5	10
Attention deficit hyperactivity disorder	11	22
Personality disorders	5	10
Supported housing	7	14
Graduated from high school	42	82
Working/studying at least half-time	25	49
Medication		
SSRI	12	24
SNRI	16	31
NaSSa	13	25
Bupropion	5	10
Tricyclic antidepressants	7	14
Mood stabilisers^¶^	13	25
Stimulants^¶^	9	18
Antipsychotics‡	11	22
Olanzapine equivalent dose (mg/day, mean/SD)	4.1	2.4

	Mean	SD
Age (years)	29.4	9.4
BMI (kg/m^2^)	26.0	6.3
CAINS score (live)	29.2	7.7
Motivation and pleasure subscale	21.4	5.9
Expression subscale	7.7	3.9
BPRS total score (video)	46.6	6.3
Negative symptoms subscale^†^	7.9	3.3
Positive symptoms subscale^†^	3.4	0.9
Affective symptoms subscale^†^	12.4	2.9
Anxiety and Tension items	6.6	2.2
Maudsley Staging Method score	11.8	12.6
CGI score	4.9	0.8
MADRS-S total	29.9	7.7
MADRS-S anhedonia*	7.4	2.4
MADRS-S anxiety*	3.6	1.3
EQ-VAS score	34.4	15.7
Sheehan Disability Scale score	17.5	7.5
AUDIT score	4.7	4.1
Animal Naming Test**	23.9	6.3
Digit Symbol Substitution Test***	50.5	18.2

^¶^Mood stabilisers included lithium, lamotrigine and valproate. Stimulants included methylphenidate, dexamphetamine, atomoxetine and guanfacine

^‡^Antipsychotics included flupentixol, quetiapine and aripiprazole

^†^Negative Symptoms Subscale comprises the items Blunted affect, Emotional withdrawal and Motor retardation. Positive Symptoms Subscale comprises the items Suspiciousness, Hallucinations and Unusual thought content. Affective Symptoms Subscale comprises the items Depression, Suicidality and Guilt.

*MADRS-S anhedonia comprises the items Lassitude and Inability to feel. MADRS-S anxiety comprises the item Inner tension

**Number of animals named during one minute

***Number of correct answers during two minutes

### Inter-rater agreement

Regarding the inter-rater agreement between live and video interviews, all ICCs were also high (all above 0.80). The ICC for the CAINS total score was 0.93 (95% CI 0.88–0.96). For the CAINS-MAP, the ICC was 0.98 (95% CI 0.96–0.99). The CI for expressional items was wider (CAINS-EXP ICC 0.81, 95% CI 0.67–0.89). For the BPRS, the ICC for the negative symptoms subscale was 0.81 (95% CI 0.67–0.89) and that for the affective symptoms subscale was 0.94 (95% CI 0.89–0.96). The ICC for the CGI was somewhat lower (ICC 0.71, 95% CI 0.50–0.84). See Additional file 1: Table S1 for all ICCs.

### Internal consistency

Cronbach’s alpha for the CAINS was 0.75 (95% CI 0.64–0.84), that for the CAINS-MAP was 0.71 (95% CI 0.57–0.82) and that for the CAINS-EXP was 0.86 (95% CI 0.79–0.92). The item-total correlation ranged from 0.32 to 0.46. All internal consistency data are presented in Table 2.

**Table 2:** Internal consistency of the individual CAINS items (*n* = 51)

		Mean value (SD)	Item–total correlation	Cronbach’s α if item deleted
Item 1	Social, family relationships	1.92 (1.04)	0.34	0.74
Item 2	Social, friendships	2.08 (0.80)	0.32	0.75
Item 3	Social, past-week pleasure	2.02 (1.30)	0.32	0.75
Item 4	Social, expected pleasure	2.41 (1.39)	0.32	0.75
Item 5	Vocational, motivation	2.63 (1.13)	0.35	0.74
Item 6	Vocational, expected pleasure	3.12 (1.41)	0.32	0.75
Item 7	Recreation, motivation	2.53 (0.88)	0.42	0.74
Item 8	Recreation, past-week pleasure	2.73 (0.92)	0.41	0.74
Item 9	Recreation, expected pleasure	1.98 (1.58)	0.41	0.74
Item 10	Expression, facial	2.22 (1.10)	0.46	0.73
Item 11	Expression, vocal prosody	1.77 (1.21)	0.46	0.73
Item 12	Expression, gestures	2.16 (1.12)	0.42	0.74
Item 13	Expression, speech	1.57 (1.19)	0.46	0.73

*CAINS* Clinical Assessment Interview for Negative Symptoms, *SD* standard deviation

### Construct validity

Construct validity measures are presented in Table 3. The CAINS total score correlated significantly with both the blinded and the non-blinded BPRS negative symptoms subscale, but more strongly with the latter; there was a significant difference between correlations (*z* = 4.82, *p* < 0.001). A similar result was seen for the CAINS-EXP, with a significant difference between correlations (*z* = 9.08, *p* < 0.001), where the strongest correlation was with the non-blinded BPRS negative symptoms subscale. There was no significant correlation between the CAINS-MAP and the BPRS negative symptoms subscale and no significant difference between correlations (*z* = 1.23, *p* = 0.22).

**Table 3:** Construct validity of the CAINS (*n* = 51)

	CAINS total Live rating	CAINS-MAP Live rating	CAINS-EXP Live rating
BPRS, video rating (blinded)^**¶**^ Negative symptoms subscale^†^	0.35 (*p* = 0.011)	0.09 (*p* = 0.510)	0.56 (*p* < 0.001)
BPRS, live rating (not blinded)^**‡**^ Negative symptoms subscale^†^	0.55^a^ (*p* < 0.001)	0.16 (*p* = 0.260)	0.84^a^ (*p* < 0.001)

Pearson correlation coefficients (*p-values*)

*CAINS* Clinical Assessment Interview for Negative Symptoms, *MAP* Motivation and Pleasure Subscale, *EXP* Expression Subscale, *BPRS* Brief Psychiatric Rating Scale

^¶^Different rater than the CAINS rating

^‡^Same rater as the CAINS rating

^†^Negative Symptoms Subscale comprises the items blunted affect, emotional withdrawal and motor retardation

^a^Indicates that the correlation coefficient is statistically different from that for the blinded video rating

### Correlations with affective domains and related measures

Correlations with affective domains are presented in Table 4. Only small and non-significant differences between the blinded versus non-blinded values of the BPRS and the CAINS were seen (data not shown), for which reason only the non-blinded values are presented. There were medium to strong correlations between the CAINS-MAP and the BPRS affective symptoms subscale, the MADRS-S total score and the MADRS-S anhedonia score, respectively. No significant correlations were found between the BPRS affective symptoms subscale and the CAINS total score or the CAINS-EXP. Both the MADRS-S total score and the MADRS-S anhedonia score correlated weakly to moderately with the CAINS total score. The MADRS-S total score did not correlate significantly with the CAINS-EXP. No significant correlations were found between either of the CAINS subscales and the MADRS-S anxiety symptoms or the BPRS anxiety symptoms. The MSM correlated significantly with the CAINS-EXP, but not with the CAINS-MAP. Both CAINS subscales correlated significantly, to a moderate or high degree, with the CGI. The EQ-VAS scores correlated significantly with the CAINS-MAP, but not with the CAINS-EXP. No significant correlations were found between the CAINS score and the SDS score. There was a weak but significant correlation between verbal fluency and the CAINS-EXP. This seemed to originate from correlations between verbal fluency and item 12 *Expressive gestures* (*r* = -– 0.35, *p* = 0.013) and item 13 *Quantity of Speech* (*r* = -– 0.31, *p* = 0.029). There were no correlations with the DSST.

**Table 4:** Correlations with affective domains and related measures (*n* = 51)

	CAINS total Live rating	CAINS-MAP Live rating	CAINS-EXP Live rating
BPRS, live rating (not blinded)^**‡**^ Affective symptoms subscale^†^	0.26 (*p* = 0.063)	0.39 (*p* = 0.005)	− 0.07 (*p* = 0.634)
MADRS-S total score	0.34 (*p* = 0.015)	0.50 (*p* < 0.001)	− 0.08 (*p* = 0.577)
MADRS-S anhedonia (Lassitude and Inability to feel)	0.29 (*p* = 0.039)	0.44 (*p* = 0.001)	− 0.08 (*p* = 0.567)
BPRS, live rating (not blinded)^**‡**^ Anxiety and Tension items	0.16 (*p* = 0.253)	0.22 (*p* = 0.113)	− 0.02 (*p* = 0.919)
MADRS-S Inner tension	0.13 (*p* = 0.358)	0.24 (*p* = 0.095)	− 0.10 (*p* = 0.504)
Maudsley Staging Method	0.22 (*p* = 0.115)	0.09 (*p* = 0.515)	0.30 (*p* = 0.031)
CGI^‡^	0.64 (*p* < 0.001)	0.45 (*p* < 0.001)	0.60 (*p* < 0.001)
EQ-VAS	− 0.27 (*p* = 0.052)	− 0.33 (*p* = 0.018)	− 0.05 (*p* = 0.744)
Sheehan Disability Scale	− 0.19 (*p* = 0.182)	− 0.22 (*p* = 0.129)	− 0.05 (*p* = 0.714)
Verbal fluency	− 0.27 (*p* = 0.059)	− 0.12 (*p* = 0.386)	− 0.34 (*p* = 0.014)
Digit Symbol Substitution Test	− 0.14 (*p* = 0.338)	− 0.09 (*p* = 0.542)	− 0.14 (*p* = 0.329)

Pearson correlation coefficients (*p-values*)

*CAINS* Clinical Assessment Interview for Negative Symptoms, *MAP* Motivation and Pleasure Subscale, *EXP* Expression Subscale, *BPRS* Brief Psychiatric Rating Scale, *MADRS-S* Montgomery Asberg Depression Rating Scale-Self Rating, *CGI* Clinical Global Impression, *EQ-VAS* EuroQol Group Visual Analogue Scale

^‡^Same rater as the CAINS rating

^†^Affective Symptoms Subscale comprises the items Depression, Suicidality and Guilt

## Discussion

In this study of negative symptoms in patients with depressive disorder, we examined the reliability and validity of the CAINS. We also explored the relationships between negative symptoms and affective symptoms. Our main result indicated that the inter-rater agreement was acceptable, which is in accordance with previous studies in schizophrenia [17]. We found the Cronbach’s alpha of the CAINS to be 0.75. This has previously been regarded as an acceptable level of internal consistency [17], though a higher level would be preferable if firm conclusions are to be drawn about the assessment of negative symptoms in depressive disorders. Our recently published findings from a study with the same design found the Cronbach’s alpha to be 0.84 in a group of 34 patients with schizophrenia [45]. Interestingly, the Cronbach’s alpha for the expression subscale CAINS-EXP was higher in our sample of patients with depressive disorder (0.86) than in the aforementioned study of patients with schizophrenia (0.75). The patients in the current study presented a mean CAINS total score of 29.2, compared with 22.0 in the schizophrenia study. In another study of the CAINS in a sample of 22 patients with depression, the mean value of the total score was 11.9 [14]. This difference probably reflects that our sample was recruited for a study of treatment of negative symptoms, with an inclusion criterion being exhibiting a certain level of self-assessed negative symptoms.

### Validity

Regarding validity, both the CAINS total score and the CAINS-EXP correlated significantly with the BPRS Negative Symptoms Subscale, regardless of blinding. However, the non-blinded results correlated more strongly. The differences between the blinded results and the non-blinded results might reflect the difficulties in obtaining high inter-rater reliability for expressional items. In other words, the blinding might not in itself be the cause of the lower coefficients for the blinded values, but rather that the second rater watched a video as opposed to a live interview, or that the values will always differ more when using two different raters for two interviews than when using the same rater for both interviews. However, a blinded study design is still recommended [42]. The CAINS-MAP did not correlate significantly with the BPRS Negative Symptoms Subscale. These results are rather logical, as both the CAINS-EXP and the BPRS negative symptoms subscale are observational measures, while the CAINS-MAP is based on a patient’s reported experience. Notably, the BPRS negative symptoms subscale contains no measures of motivation and/or experienced pleasure. In our recent similar study of patients with schizophrenia, the correlation coefficient between the CAINS-EXP and the BPRS Negative Symptoms Subscale was 0.48 [45]. Given the correlation coefficient of 0.56 for the depressed population in our study, it can be argued that the CAINS-EXP might be well-suited for rating negative symptoms also in patients with depression.

### Negative and affective symptoms domains

When analysing relationships between negative symptoms and affective symptoms, we found moderate correlations between the CAINS-MAP and the BPRS Affective Symptoms Subscale, as well as between the CAINS-MAP and the MADRS-S total score. Given the suggested overlap between depressive and negative symptoms, we do not regard this as surprising [14]. Some validation studies of the CAINS in patients with schizophrenia found only weak and non-significant correlations between the CAINS and the Calgary Depression Scale for Schizophrenia (CDSS) [17, 46, 47], whereas others found significant associations [48]. Regarding the correlation with MADRS-S, our findings in patients with schizophrenia resulted in a correlation coefficient of 0.67, i.e. even stronger than the 0.50 in the current study. Notably, it seems that it was mainly the anhedonia items of the MADRS-S that contributed to this correlation. We are not aware of any other study comparing the CAINS and the MADRS-S. The negative symptoms construct has been suggested to reflect an entity distinct from depressive symptoms [5]. However, our results support the notion that there is an overlap between negative symptoms and depressive symptoms [14]. Yet, this overlap is not total, which is supported by our findings that the CAINS-EXP is more strongly correlated to the BPRS negative symptoms subscale than to the BPRS affective symptoms subscale. Also the CAINS-EXP did not correlate to MADRS-S. These results indicate that negative and depressive symptoms are indeed different constructs, albeit being partly overlapped. To further distinguish the negative symptoms in schizophrenia from depressive symptoms, more research is needed to establish which features can differentiate between schizophrenia and depressive disorders. There have been suggestions that expressional items might constitute such a feature [14]. One study that argued for a clearly distinct negative symptom cluster used the Positive and Negative Syndrome Scale [49] and the CDSS. These scales may be more suited for distinguishing between the disorders. Even if reliance on expressional features as a basis for distinction is a feasible way forward in a clinical setting, our study does not lend support to this notion, as our sample of patients with depression exhibited scores on the CAINS-EXP comparable to those in some studies of schizophrenia [46, 48, 50]. However, in this study we studied only patients with depression, and further studies could merit from including transdiagnostic samples. There is also a need to investigate symptom levels in the general population, especially of the volitional items, in order to establish a more accurate and valid pathological construct. Some research has been conducted on this topic [51]. However, from a clinical perspective, the CAINS might constitute a valuable tool in the assessment of the depressive symptom burden. Notably, some contents of the CAINS are lacking in most established symptom rating scales for depression, for example the purely behavioural items. Focusing on such items might be helpful for patients with activation agendas, for instance.

None of the anxiety measures correlated with the CAINS. As assessments of anxiety are lacking in most validation studies of the CAINS, it is difficult to compare this result to others. None of the items in the CAINS explicitly address feelings of worry or anxiety, for which reason we do not regard the lack of an association as surprising.

### Other measures

We observed a weak correlation between the MSM and the CAINS-EXP. There are many ways to define treatment-resistant depression, and our finding could be an argument for using a continuous measure rather than a dichotomous one. Though the correlation was weak, this finding also suggests that expressional factors can be linked to increased levels of treatment resistance in depression. Further, there was a moderate correlation with the CGI, indicating that expressional deficits are important for the impression of severity in the global clinical picture. These results were virtually identical to our findings in the population with schizophrenia [45]. For self-assessed health, there was a weak correlation with the CAINS-MAP only, reflecting that patients who experience decreased motivation and pleasure will rate their overall health as worse. This indicates that motivational deficits and anhedonia are connected to something that matters to patients, which should encourage further exploration. The CAINS has been associated with functioning measures in schizophrenia [17, 50, 52], although we did not replicate this finding in our study of schizophrenia [45]. In the current study of patients with depression, we did not find a correlation between functioning (as assessed with the SDS) and CAINS total score. This might have many reasons, but it is known that functional deficits are not only explained by symptom severity [53]. The weak correlation between verbal fluency and the CAINS-EXP most likely reflects mainly the *Quantity of speech* item. Apart from that, our results are similar to those of other studies using the CAINS in schizophrenia, i.e. the CAINS does not seem to reflect cognitive functioning only.

### Limitations

Some limitations of the current study need to be addressed. The small and heterogenous sample size prevents drawing firm conclusions and our findings need to be replicated in larger samples. The sample size also precludes factor structure analyses. A sample size calculation was performed in the original treatment study from where the current sample was recruited [20]. Since our sample consisted of patients with treatment-resistant depression, the results might not be generalizable to a wider population of patients with depressive disorders. Indeed, our patients were recruited to a treatment study of negative symptoms, and one of the inclusion criteria was exhibiting a certain level of self-rated negative symptoms. Nevertheless, as the correlation between our measure of treatment resistance and CAINS total score was weak, there is room for less treatment-resistant patients to exhibit negative symptoms. The high comorbidity with anxiety disorders is a rule rather than an exception in depression [54] and does not limit generalizability to the same extent. Further, anxiety did not correlate with CAINS total score, indicating that the presence of an anxiety disorder is of limited importance here. Around one-fifth of the patients had a diagnosis of attention deficit hyperactivity disorder, which should be kept in mind when assessing generalizability. A wide range of psychopharmacological agents was used by the study participants. Antipsychotics, which were prescribed to around one-fifth of the sample, are of some relevance for negative symptoms. However, the olanzapine equivalent dose was rather low. Thus, the antidopaminergic effect probably did not contribute to negative symptoms to any greater extent. Although the training procedures resulted in an acceptable level of inter-rater agreement (all ICCs above 0.80), we acknowledge that there might still be a qualitative difference between rating a participant’s score during a live interview as opposed to a video recording, and maybe especially so for the expressional items. This might contribute to the differences noted between the live and video ratings in Table 3. Lastly, adding other scales for negative symptoms and/or anhedonia could have strengthened the study. The BPRS negative symptoms subscale might not capture the full range of negative symptoms domains, but it has nevertheless been used in many validation studies. Most notably, the domains of asociality and avolition might not be entirely covered by the BPRS negative symptoms subscale. The Positive and Negative Syndrome Scale [49] and the Scale for the Assessment of Negative Symptoms [55] are also widely used. The negative symptoms construct is ultimately defined by the rating scale used and will therefore always differ somewhat across studies. The CAINS was constructed to deal with some of the shortcomings observed in earlier scales and is supposed to have better face validity [1].

### Clinical utility

Negative symptoms are burdensome for the patients, and often also implicate hardships in relation to peers and counsellors. The notion that patients with depression also exhibit negative symptoms call for attention in clinical practice. Our experience from interviewing patients with depression with the CAINS is that both patients and their relatives appreciated the slightly different questions than what they were used to be asked. The feedback given was often that the questions addressed key issues regarding their behaviour and function, which they found valuable and accurate.

## Conclusions

Assessment of negative symptoms in depression can be performed with the CAINS, with good inter-rater agreement and acceptable internal consistency and validity. There are associations between negative and depressive symptoms but the overlap is not total, thus strengthening the notion of two partly different constructs. To more accurately define the negative symptoms construct, we encourage further use of the CAINS in populations with depressive disorders as well as direct comparisons between patients with depression and patients with schizophrenia.

### Supplementary Information

**Additional file 1: Table S1.** Inter-rater agreement after training and between live and video ratings.

## Data Availability

The datasets used and/or analysed during the current study are available from the corresponding author on reasonable request.

## Abbreviations

AUDIT: Alcohol Use Disorders Identification Test
BPRS-E: Brief Psychiatric Rating Scale Expanded
CAINS: Clinical Assessment Interview for Negative Symptoms
CAINS-EXP: CAINS Expression Subscale
CAINS-MAP: CAINS Motivation and Pleasure Subscale
CGI: Clinical Global Impression
CI: Confidence interval
DSST: Digit Symbol Substitution Test
EQ-VAS: EuroQol Group Visual Analogue Scale
ICC: Intraclass correlation coefficient
MADRS-S: Montgomery-Asberg Depression Rating Scale
MAP-SR: Motivation and Pleasure Scale-Self Report
MSM: Maudsley staging method
SD: Standard deviation
SDS: Sheehan Disability Scale
