# Supplementary material for: Psychometric properties of the Clinical Assessment Interview for Negative Symptoms (CAINS) in patients with depression and its relationship to affective symptoms
Source: Ann Gen Psychiatry. 2023 Oct 26;22:42. doi: 10.1186/s12991-023-00474-x (PMC10604520; doi:10.1186/s12991-023-00474-x)
Supplement: Supplementary file 1 — Additional file 1: Table S1. Inter-rater agreement after training and between live and video ratings. [file 12991_2023_474_MOESM1_ESM.docx]

**Additional file 1: Table S1.** Inter-rater agreement after training and between live and video ratings.

|  | After training  (*n*=9) | Live versus video (*n*=51) |
| --- | --- | --- |
|  | ICC (95% CI) | ICC (95% CI) |
| CAINS, total score | 0.97 (0.91–0.99) | 0.93 (0.88–0.96) |
| Motivation and pleasure subscale | 0.99 (0.97–1.00) | 0.98 (0.96–0.99) |
| Expression subscale | 0.86 (0.60–0.96) | 0.81 (0.67–0.89) |
| BPRS, total score | 0.94 (0.84–0.99) | 0.90 (0.83–0.94) |
| Negative symptoms subscale^†^ | 0.82 (0.50–0.96) | 0.81 (0.66–0.89) |
| Positive symptoms subscale^†^ | 0.95 (0.85–0.99) | 0.87 (0.77–0.92) |
| Affective symptoms subscale^†^ | 0.98 (0.95–1.00) | 0.94 (0.89–0.96) |
| Anxiety and Tension items | 0.91 (0.74–0.98) | 0.80 (0.83–0.94) |
| CGI | 0.73 (0.25–0.93) | 0.71 (0.50–0.84) |

ICC = Intraclass correlation coefficient, CI = Confidence interval, CAINS = Clinical Assessment Interview for Negative Symptoms, BPRS = Brief Psychiatric Rating Scale, CGI = Clinical Global Impression.

^†^Positive symptoms subscale comprises the items Suspiciousness, Hallucinations and Unusual thought content. Negative symptoms subscale comprises the items Blunted affect, Emotional withdrawal and Motor retardation. Affective symptoms subscale comprises the items Depression, Suicidality and Guilt.
